# NH_3_ molecular doping of silicon nanowires grown along the [112], [110], [001], and [111] orientations

**DOI:** 10.1186/1556-276X-7-308

**Published:** 2012-06-18

**Authors:** Álvaro Miranda, Xavier Cartoixà, Enric Canadell, Riccardo Rurali

**Affiliations:** 1 Institut de Ciència de Materials de Barcelona (ICMAB-CSIC), Campus de Bellaterra, BellaterraBarcelona, 08193, Spain; 2 Departament d’Enginyeria Electrònica, Universitat Autònoma de Barcelona, Campus de Bellaterra, Bellaterra, Barcelona, 08193, Spain; 3 Instituto Politécnico Nacional, ESIME-Culhuacan, Av. Santa Ana 1000, México D.F., 04430, México

**Keywords:** Silicon nanowires, Ammonia, Molecular doping, DFT, Electronic properties, Gas sensing

## Abstract

The possibility that an adsorbed molecule could provide shallow electronic states that could be thermally excited has received less attention than substitutional impurities and could potentially have a high impact in the doping of silicon nanowires (SiNWs). We show that molecular-based *ex-situ* doping, where NH_3_ is adsorbed at the sidewall of the SiNW, can be an alternative path to *n*-type doping. By means of first-principle electronic structure calculations, we show that NH_3_ is a shallow donor regardless of the growth orientation of the SiNWs. Also, we discuss quantum confinement and its relation with the depth of the NH_3_ doping state, showing that the widening of the bandgap makes the molecular donor level deeper, thus more difficult to activate.

## Background

Semiconductor nanowires exhibit a variety of unique material properties, including mechanical flexibility, size-dependent optical and electronic properties, and solution processability. In particular, silicon nanowires (SiNWs) have been explored and studied both theoretically [1] and experimentally [2] for a long time, and they have attracted much attention for many applications, such as bipolar and field-effect transistors [3-5], nanosensors [3,6], solar cells [7,8], and energy conversion devices [9,10], but controlled doping with electronic and magnetic impurities remains an important challenge [11-14]. While developing these applications, it is important to control the electrical and optical properties of nanowires (NWs), which strongly depend on the diameter as well as the crystallographic orientation [15] and defect structure of the NWs [16]. There are several ways that the electronic structure of SiNWs can be modified, for example, by changing the thickness, orientation, surface morphology, hydrogen concentration, and doping [1,2]. Reduced-dimensionality systems are characterized by a large surface-to-bulk ratio and offer the possibility of doping through the external adsorption of molecules [17] rather than the incorporation of substitutional impurities [18].

The increase in graphene carrier mobility induced by absorbed gas molecules has been recently used as a highly sensitive solid-state sensor capable of detecting individual molecules [17,19]. This has motivated molecular doping calculations in graphene [20,21], where the vanishing bandgap makes it easier to find molecular adsorbates whose HOMO (LUMO) falls in the host conduction (valence) band. Also, adsorption of NH_3_ and NO_2_ has been predicted to occur in carbon nanotubes [22], where charge transfer and gas-induced charge fluctuations should affect significantly the transport properties of single-walled carbon nanotubes [23], in agreement with the experimental results of Kong [24].

In SiNWs, the possibility that an adsorbed molecule could provide shallow electronic states has received less attention, though some promising experimental results have been recently reported [25,26]. This is relevant because traditional substitutional dopants in Si have too large activation energies in thin SiNWs [27,28]. On the other hand, many experimental results have been obtained with mesoporous Si (meso-PSi) [29-31], where electrochemical attack of a Si sample yields a disordered network of single-crystalline Si wires. In the work of Garrone et al. [32], *n*- and *p*-type doping was achieved by the exposure of meso-PSi to NH_3_ and NO_2_, respectively.

Some of us have recently studied the chemisorption of NH_3_ and NO_2_ on SiNWs [33], reporting *n*- and *p*-type doping of a SiNW with dangling bonds upon gas exposure. That work, however, was restricted to the {110} facet of a [111] SiNW. Focusing on NH_3_ the present work will show that those conclusions can be extended to SiNWs grown along other low index orientations, with the molecule sticking to facets of any crystallographic orientation. We will also consider the effect of the diameter of the NW since it is not obvious whether the impurity state introduced by the dopant molecule will be subject to the effects of quantum confinement.

## Methods

**Figure 1:**
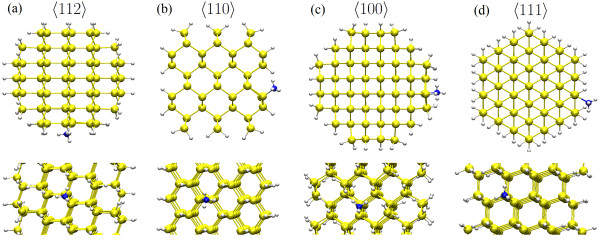
**Structures of the adsorption of NH_3_ on SiNWs.** Viewed from top (upper) and side {110}(lower): (**a, b, c, d**) the SiNWs grown along the [112], [110], [001], and [111] directions, respectively. Yellow, white, and blue spheres indicate Si, H, and N atoms, respectively.

All of our *ab initio* calculations were performed with the Siesta code [34], which implements density functional theory (DFT). We use norm-conserving pseudopotentials for the core electrons and expand the one-electron wane function of the valence electron with a double-*ζ*basis set plus polarization functions [35]. The exchange correlation energy is calculated within the generalized gradient approximation (GGA) in the parametrization of Perdew-Burke-Ernzerhof [36]. We consider silicon nanowires hydrogenated with a diameter of approximately 1.5 nm grown along the [112],[110],[001], and [111] directions, common growth orientations that have been observed experimentally to date [37,38]. The geometrical structures of freestanding NWs are shown in Figure 1 and have been relaxed until all the forces were lower than 0.04 eV/Å. We study NH_3_ molecular doping of silicon nanowires in the [112],[110],[001], and [111] directions, using supercells made of three, five, three, and two primitive cells, respectively, to allow neglecting the spurious interaction with the periodic image of the molecule and to obtain converged results of the total energies [39]. Also, we study the effect of quantum confinement of NH_3_ chemisorbed on 1.0-, 1.5-, and 2.0-nm-thick [111] SiNWs, supercells made of three primitive cells. The Brillouin zone has been sampled with a 1×1×2 grid of *k*-points within the Monkhorst-Pack algorithm [40].

## Results and discussion

We study NH_3_ adsorption on 1.5-nm SiNWs grown along the [112],[110],[001], and [111] orientations (see Figure 1) as well as at different facets such as {110}, {100}, and {111}. Like in the case of Miranda-Durán et al. [33], we focus on the chemistry of the dangling bond (DB)-molecule complex, trying to assess a possible dependence on the facet orientation, considering this as the most effective adsorption mechanism.

We have found that all the adsorption processes considered are favored, with the N bonding the unpaired electron of the Si DB. Chemisorption energies are shown in Table 1, from which we see that NH_3_ molecules will always bind to available dangling bonds. Subsequent desorption at room temperature will be substantially suppressed, as binding energies are, at least, of the order of 8_*kB*_*T*, with {110} facets being the ones providing the most stable binding.

**Table 1:** Chemisorption energies of adsorption NH_3_ on SiNWs to different facets

**Growth orientations**	**Bandgap**	**Facet *{*110*}***	**Facet *{*100*}***	**Facet *{*111*}***
[112]	1.76, indirect	0.40	N/A	0.21
[110]	1.41, direct	0.33	0.30	N/A
[001]	1.89, direct	0.33	0.22	N/A
[111]	1.78, indirect	0.24	N/A	N/A

**Figure 2:**
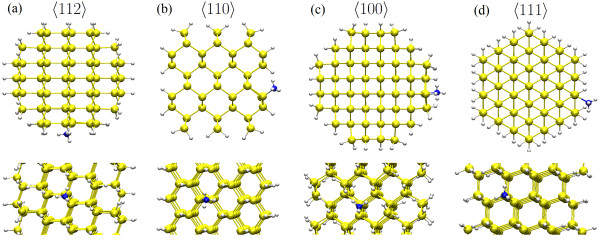
**Electronic band structure and projected DOS with NH_3_ adsorbed at a {110} facet.** Electronic band structures, NH_3_ adsorption yield *n*-type doping, pining the Fermi level at the bottom of the conduction band (left panels) for SiNWs along the (**a**) [112], (**b**) [110], (**c**) [001], and (**d**) [111] directions. The projected DOS (right panels) illustrates the contribution of the molecular orbitals.

**Figure 3:**
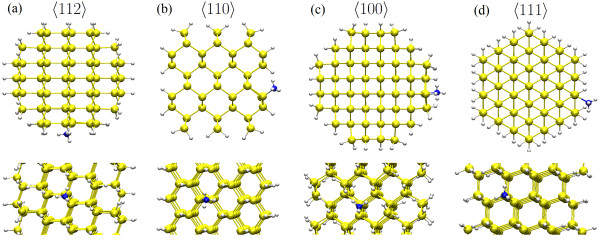
**Structures of the adsorption of NH_3_ on SiNWs.** Viewed from top (upper) and sidewalls other than the {110} facetside (lower): (**a, b, c**) the SiNWs grown along the [112], [110], and [001] directions, respectively. Yellow, white, and blue spheres indicate Si, H, and N atoms, respectively.

**Figure 4:**
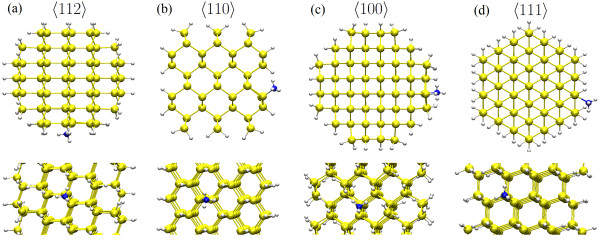
**Electronic band structure and projected DOS with NH_3_ adsorbed at facets other than {110}.** Electronic band structures, NH_3_ adsorption yields n-type doping, pining the Fermi level at the bottom of the conduction band (left panels) for SiNWs along the (**a**) [112], (**b**) [110], and (**c**) [001], directions. The projected DOS (right panels) illustrates the contribution of the molecular orbitals.

In Figure 2, we plot the band structure corresponding to the adsorption of an NH_3_ molecule at the DB of the {110} facet for all the growth orientation considered. The formation of the DB-molecule complex yields a shallow molecular state close to the conduction band edge, thus resulting in n-type doping, similar to what we previously reported [33]. Therefore, NH_3_ is found to be an efficient donor, with carriers that can be thermally excited into the conduction band. The efficiency of the doping process is, at first approach, independent on the wire growth orientation. Band structures of the less favored, but possible, adsorption mechanisms, i.e., at sidewalls other than the {110} facet (Figure 3), are shown in Figure 4. The conclusions are qualitatively the same, although in the cases shown, the molecular state is somewhat deeper than for the adsorption at {110} facets. Shallower states will be recovered at slightly larger diameters (see the discussion on quantum confinement below). These results suggest that NH_3_ can act as a donor agent, a conclusion also corroborated by experimental results [32]. It should be kept in mind that SiNWs in this size range, for instance, have been shown to have calculated bandgaps within GGA that are 0.6 to 0.8 eV smaller than GW or DFT hybrid functional calculations [41,42], which are close to experimental results [37]. Also, as discussed recently by Niquet et al. [43], density functional calculations, at least within the local and semilocal approximations to the exchange-correlation functionals, only allow a qualitative inspection of this kind of systems, and a many-body treatment is required for a quantitative estimation of the dopant binding energy. For these reasons, it cannot be guaranteed that the estimation of the molecular state depth is quantitatively accurate, and beyond DFT calculations are needed to clarify this point. Therefore, the results presented here should be taken to be qualitative.

**Figure 5:**
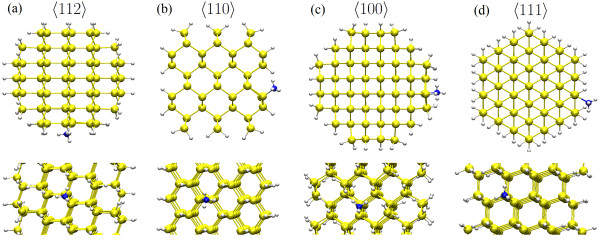
**Adsorption of NH_3_ on the {110} face of the SiNWs along the [111] direction Viewed from top (upper) and side (lower): (**a**) 1.**0, (**b**) 1.5, (**c**) 2.0 the SiNWs along the [111] direction. Yellow, white, and blue spheres indicate Si, H, and N atoms, respectively.

**Figure 6:**
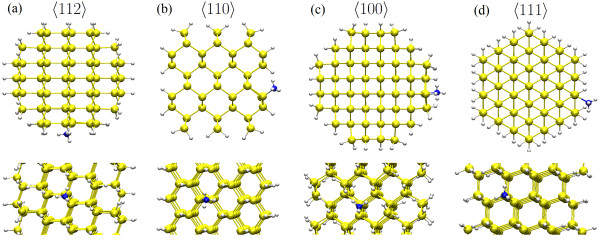
**Electronic band structure and projected DOS for NH_3_ adsorption on a [111]-oriented SiNW Electronic band structures (left panels), showing a deep state, a donor shallow state close to the conduction band edge and a donor state fell inside the conduction band for (**a**) 1.**0-, (**b**) 1.5-, and (**c**) 2.0-nm-thick SiNWs, respectively. The projected DOS (right panels) illustrates the contribution of the molecular orbitals.

Next, we study the effect of the wire diameter. For this, we have considered NH_3_ adsorption on three different hydrogenated SiNWs with diameters of 1.0, 1.5, and 2.0 nm grown along the [111] orientation and bounded by {110} facets (see Figure 5). These small diameters yield a sizeable quantum confinement effect [41], which leads to the widening of the electronic bandgap. The electronic band structures of the adsorbed configurations of Figure 5 are shown in Figure 6. In the smaller diameter SiNW (Figure 5a), with a bigger bandgap, NH_3_ adsorption yields a deep state, as illustrated by Figure 6a. On the other hand, we obtain for the 1.5-nm NW that the adsorbed molecule contributes with a localized shallow state close to the conduction band edge, where it pins the Fermi level (see Figure 6b), similar to the case of conventional substitutional dopants for bulk Si, such as P. In the limit of a large diameter SiNW, the NH_3_ donor state falls at the conduction band edge, as shown in Figure 6c, thus confirming that in larger NWs, where the bandgap is smaller due to a reduced quantum confinement; this state is shallow enough to be an active electron donor.

## Conclusions

In summary, we have reported electronic structure calculations of the adsorption of NH_3_ onto SiNWs grown along the [112],[110],[001], and [111] orientations, considering adsorption at dangling bonds located at {110}, {100}, and {111} facets, where applicable. We recover the main feature reported in the experiments that NH_3_ is a donor in nanostructured Si, extending the conclusions reported in the study of Miranda-Durán et al. [33], where only one specific adsorption configuration was considered. We have found that NH_3_ is more strongly bound to {110} facets, regardless of the growth orientation of the SiNWs. On the other hand, quantum confinement effects turn the shallow impurity level into a deep one as the SiNW diameter is decreased. All these observations suggest that NH_3_ can be used as an active *n*-type dopant in thin SiNWs.

## Abbreviations

Db, dangling bond; DFT,density functional theory; GGA, generalized gradient approximation; NWs, nanowires; SiNWs, silicon nanowires.

## Competing interests

The authors declare that they have no competing interests.

## Authors’ contributions

AM carried out the calculations, participated in the sequence alignment, and drafted the manuscript. XC participated in the sequence alignment and drafted the manuscript. EC participated in the sequence alignment and drafted the manuscript. RR participated in the sequence alignment and drafted the manuscript. All authors read and approved the final manuscript.
